# Tumor reoxygenation for enhanced combination of radiation therapy and microwave thermal therapy using oxygen generation in situ by CuO nanosuperparticles under microwave irradiation

**DOI:** 10.7150/thno.42818

**Published:** 2020-03-25

**Authors:** Zengzhen Chen, Wenna Guo, Qiong Wu, Longfei Tan, Tengchuang Ma, Changhui Fu, Jie Yu, Xiangling Ren, Jianming Wang, Ping Liang, Xianwei Meng

**Affiliations:** 1 Laboratory of Controllable Preparation and Application of Nanomaterials, Key Laboratory of Cryogenics, Technical Institute of Physics and Chemistry, Chinese Academy of Sciences, No. 29 East Road Zhongguancun, Beijing 100190, People's Republic of China.; 2 Department of Nuclear Medicine, Harbin Medical University Cancer Hospital, Harbin 150086, People's Republic of China.; 3 Department of Interventional Ultrasound, Chinese PLA General Hospital, Beijing 100853, People's Republic of China.; 4 Beijing Institute of Fashion Technology. No. A2, East Yinghua Street, Chaoyang District, Beijing 102209, People's Republic of China.; 5 University of Chinese Academy of Sciences, Beijing 100049, People's Republic of China.; 6 School of Information Engineering, Inner Mongolia University of Science and Technology, Baotou 014010, People's Republic of China.

**Keywords:** reoxygenation, tumor, microwave, thermal therapy, radiation therapy

## Abstract

As known, radiation therapy (RT) can exacerbate the degree of hypoxia of tumor cells, which induces serious resistance to RT and in turn, is the greatest obstacle to RT. Reoxygenation can restore the hypoxic state of tumor cells, which plays an important role in reshaping tumor microenviroment for achieving optimal therapeutic efficacy. Herein, we report for the first time that microwave (MW)-triggered IL-Quercetin-CuO-SiO_2_@ZrO_2_-PEG nanosuperparticles (IQuCS@Zr-PEG NSPs) have been used to achieve an optimal RT therapeutic outcomes by the strategy of upregulating tumor reoxygenation, *i.e.* hypoxic cells acquire oxygen and return to normal state.

**Methods**: We prepared a promising multifunctional nanosuperparticle to upregulate tumor reoxygenation by utilizing CuO nanoparticle to generate oxygen under MW irradiation in the tumor microenvironment. The IQuCS@Zr-PEG NSPs were obtained by introducing CuO nanoparticles, MW sensitizer of 1-butyl-3-methylimidazolium hexafluorophosphate (IL), radiosensitizer of Quercetin (Qu) and surface modifier of monomethoxy polyethylene glycol sulfhyl (mPEG-SH, 5k Da) into mesoporous sandwich SiO_2_@ZrO_2_ nanosuperparticles (SiO_2_@ZrO_2_ NSPs). The release oxygen by IQuCS@Zr-PEG NSPs under MW irradiation was investigated by a microcomputer dissolved oxygen-biochemical oxygen demand detector (DO-BOD) test. Finally, we used the ^99m^Tc-HL91 labeled reoxygenation imaging, Cellular immunofluorescence, immunohistochemistry, and TUNEL experiments to verify that this unique MW-responsive reoxygenation enhancer can be used to stimulate reshaping of the tumor microenvironment.

**Results**: Through experiments we found that the IQuCS@Zr-PEG NSPs can persistently release oxygen under the MW irradiation, which upregulates tumor reoxygenation and improve the combined tumor treatment effect of RT and microwave thermal therapy (MWTT). Cellular immunofluorescence and immunohistochemistry experiments demonstrated that the IQuCS@Zr-PEG NSPs can downregulate the expression of hypoxia-inducible factor 1α (HIF-1α) under MW irradiation. The ^99m^Tc-HL91 labeled reoxygenation imaging experiment also showed that the oxygen generated by IQuCS@Zr-PEG NSPs under MW irradiation can significantly increase the reoxygenation capacity of tumor cells, thus reshaping the tumor microenvironment. The high inhibition rate of 98.62% was achieved in the antitumor experiments *in vivo*. In addition, the IQuCS@Zr-PEG NSPs also had good computed tomography (CT) imaging effects, which can be used to monitor the treatment of tumors in real-time.

**Conclusions**: The proof-of-concept strategy of upregulating tumor reoxygenation is achieved by MW triggered IQuCS@Zr-PEG NSPs, which has exhibited optimal therapeutic outcomes of combination of RT and MWTT tumor. Such unique MW-responsive reoxygenation enhancer may stimulate the research of reshaping tumor microenvironment for enhancing versatile tumor treatment.

## Introduction

RT is one of the three major clinical cancer treatment methods 1-6. It mainly uses free radicals generated by ionizing radiation (such as X-rays, γ-rays, *etc.*) to react with oxygen 7-10, which forms the peroxide free radicals (*e.g*., ROO•, R_2_HR´OO•, *etc.*). These peroxide free radicals can destroy the DNA macromolecules of cancer cells and kill cancer cells 11-13, so the treatment outcomes depend largely on the oxygen level in tumor microenvironment. Unfortunately, tumor cells is usually in a hypoxic state 14-18. Due to the rapid growth of tumor cells, the growth rate of blood vessels is relatively delayed 19, 20. These factors lead to different blood supply within the tumor, which makes the tumor cells mainly in a hypoxic state 21. Hypoxia in tumor cells can seriously impede the efficacy of RT, leading to incomplete treatment of tumors and high recurrence 22-25. Hypoxic cells acquire oxygen and gradually return to normal state, that is, reoxygenation, which is the key to clinically achieve the optical therapeutic effect of RT 26,27. It can increase the sensitivity of hypoxic cells to RT, reversing or reducing the hypoxic state of solid tumors 28. Therefore, during the RT, if hypoxic cells have the opportunity to achieve reoxygenation, this will reduces the negative effects of hypoxic cells, which will increase the killing effect of RT on the initial hypoxic cells 29,30.

Strategies for upregulating tumor reoxygenation to enhance the efficacy of RT have attracted widespread attentions in recent years. These strategies mainly include: hyperbaric oxygen therapy 31-33, oxygen-carrying nanocarriers, and O_3_ therapy. Among them, hyperbaric oxygen therapy is a single exposure of tumor cells to high concentrations of oxygen (60% O_2_, 456 mmHg), and the tumor cells undergo reoxygenation after treatment, which can significantly improve the therapeutic effect on the tumor 34,35. The oxygen-carrying nanocarrier utilizes nanoparticles as an oxygen shuttle 36-39. The oxygen is transported to the tumor area through the blood vessel, which increases the partial pressure of oxygen in the tumor tissue, thereby causing reoxygenation of hypoxic cells 40. The O_3_ therapy uses O_3_ to increase the oxygen content in the bloodstream and accelerate the reoxygenation rate, so as to solve the resistance of hypoxic cells to RT 41,42. Despite these encouraging advances, there are still some problems with strategies to improve reoxygenation by increasing oxygen partial pressure in the blood 43-45. For example, these strategies have poor selectivity for tumors and weak oxygen carrying capacity, which results in the unobvious reoxygenation of tumor cells 46-49. Therefore, it is highly desirable to develop a smart system capable of reoxygenating in tumor cells during RT, so as to eradicate tumor synergistically.

**Scheme 1:**
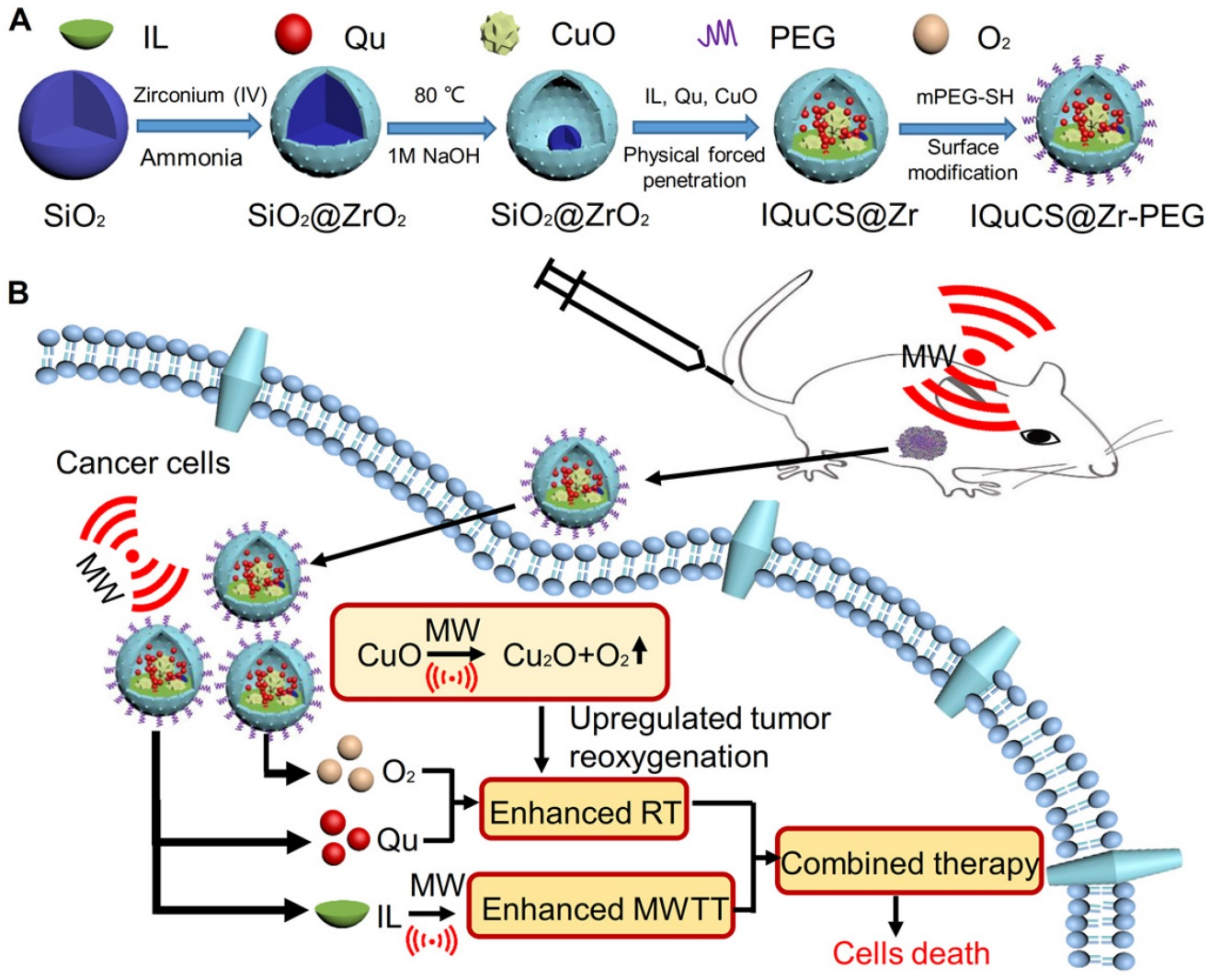
(A) A schematic diagram of the synthesis of IQuCS@Zr-PEG NSPs is not drawn to scale. (B) Schematic diagram of IQuCS@Zr-PEG NSPs with oxygen generation upregulating tumor reoxygenation for enhanced combination of radia-microwave thermal therapy by MW irradiation.

Herein, based on the principle that CuO nanoparticles can generate oxygen under MW irradiation 50, we have envisioned a strategy of reshaping the tumor microenvironment. Using CuO nanoparticles generate oxygen under MW irradiation to upregulate tumor reoxygenation, which can enhance the combined tumor treatment of RT and MWTT. Briefly, The IQuCS@Zr-PEG NSPs were obtained by introducing CuO nanoparticles, radiosensitizer of Quercetin (Qu) 51,52, MW sensitizer of 1-butyl-3-methylimidazolium hexafluorophosphate (IL) and surface modifier of monomethoxy polyethylene glycol sulfhyl (mPEG-SH, 5k Da) into mesoporous sandwich SiO_2_@ZrO_2_ nanosuperparticles (SiO_2_@ZrO_2_ NSPs) (**Scheme 1A**). The oxygen release characteristics of IQuCS@Zr-PEG NSPs under MW irradiation were studied by a microcomputer dissolved oxygen-biochemical oxygen demand detector (DO-BOD) test. It was found that the concentration of oxygen generated was 3.10 times higher than that of the bare solution (phosphate-buffered saline, PBS, 20 min after MW irradiation). Therefore, when the IQuCS@Zr-PEG NSPs were enriched in the tumor tissue, they generated a large amount of oxygen under MW irradiation and continuously released oxygen in tumor site, which significantly increased the concentration of oxygen and oxygen pressure in the tumor microenvironment. It provides sufficient conditions for the reoxygenation of tumor hypoxic cells, thereby reshaping the tumor microenvironment. And the reoxygenation process of hypoxic cells was verified by ^99m^Tc-HL91 labeled reoxygenation imaging experiments. Antitumor experiments demonstrated that the IQuCS@Zr-PEG NSPs had a very high inhibitory effect on tumor under MW irradiation and combination therapy, and the tumor inhibition rate reached into 98.62%. Moreover, cellular immunofluorescence and immunohistochemistry experiments proved that the IQuCS@Zr-PEG NSPs can downregulate the expression of HIF-1α under MW irradiation, and reflected the reoxygenation of tumor cells from the side, which maybe the key factor for the high tumor inhibition rate. In addition, the IQuCS@Zr-PEG NSPs also have good CT imaging effects, which can be used to monitor the treatment of tumors in real-time. Therefore, the as-made IQuCS@Zr-PEG NSPs can be used as a MW-responsive reoxygenation enhancer, which can obviously enhance the combined tumor treatment of CT imaging-guided RT and MWTT.

## Materials and Methods

### Materials

Zirconium (IV) propoxide was obtained from the Tokyo Chemical Industry Co., Ltd. Qu was obtained from the Harbin Medical University Cancer Hospital. IL was provided by Shanghai Chengjie Chemical Co., Ltd (China). mPEG-SH was obtained from the Beijing Kaizheng United Medical Technology Co., Ltd. Copper sulfate pentahydrate (CuSO_4_·5H_2_O) was provided by Xiqiao Chemical Co., Dissolved oxygen reagents were purchased from the Beijing Anshengdacheng Electronics Co., Ltd. Ltd. 1,4-dioxane was provided by the Beijing Chemical Factory. Ammonia was obtained from the Sinopharm Group Chemical Reagent Co., Ltd. All the reagents used in the experiments were analytical grades, and they need not be further purified when used.

### Testing and characterization

SEM (SEM-4800, Nisshin Japan) and TEM (HT7700, Nisshin Japan) were used to characterize the size and morphology of SiO_2_ nanoparticles, SiO_2_@ZrO_2_ NSPs and CuO-SiO_2_@ZrO_2_ nanosuperparticles (CuO-SiO_2_@ZrO_2_ NSPs). DO-BOD (Hanaward (Beijing) Instrument Co., Ltd.) was used to quantify determination of oxygen concentration in solution under MW irradiation. UV-Vis (Varian) and FTIR (model 3100 Excalibur) were used to characterize the surface functional groups of the IQuCS@Zr-PEG NSPs. The temperature changes under the MW irradiation in this work were monitored by an FLIR system. Enzyme labelling apparatus (Thermo Fisher Instruments, Inc.) was used to detect the cell viability. The H&E staining photos was employed the confocal fluorescence microscopy (Olympus X71, Japan) to observe.

### Animals

In this experiment, we conducted animal experiments in strict accordance with the relevant provisions of the eighth edition (International Publication No.: 978-0-309-15400-0) Guide for the Care and Use of Laboratory Animals, which were approved by Institutional Animal Care and Use Committee (IACUC) of the Harbin Medical University Cancer Hospital Animal Care and Use Committee.

BALB/c nude mouse (16±1g), were raised at 25±1 °C and 50-53% humidity at the Experimental Animal Center of Harbin Medical University. We subcutaneously injected 2×10^6^ human lung adenocarcinoma A549 cells in 100 μL PBS into abdomen of each BALB/c nude mouse to establish the A549 tumor bearing model.

### Preparation of the IQuCS@Zr-PEG NSPs

We synthesized the IQuCS@Zr-PEG NSPs in four steps. Firstly, mesoporous sandwich SiO_2_@ZrO_2_ NSPs were synthesized using SiO_2_ nanoparticles as templates. After that, CuO nanoparticles were encapsulated into SiO_2_@ZrO_2_ NSPs to prepare the CuO-SiO_2_@ZrO_2_ NSPs. Then, the IQuCS@Zr NSPs were prepared by introducing the IL and the radiosensitizer of Qu. Finally, mPEG-SH was used for surface modification of the IQuCS@Zr NSPs to obtain the IQuCS@Zr-PEG NSPs with low toxicity. The detailed preparation methods were as follows.

#### Preparation of SiO_2_@ZrO_2_ NSPs

Mesoporous sandwich SiO_2_@ZrO_2_ NSPs were prepared by using the solid SiO_2_ nanoparticles as templates. 2 mL of solid SiO_2_ nanoparticles ethanol solution (approximately 210 mg), 1.3 mL of ammonia (NH_3_·H_2_O, 25-28%), 150 mL of ethanol and 50 mL of acetonitrile (acetonitrile/ethanol=1:3) were added into a conical flask (250 mL) to mix evenly. After that, the 0.6 mL of the Zirconium (IV) propoxide was poured in the mixed solution. After stirring for 8 h at room temperature, Zirconium (IV) propoxide was slowly hydrolyzed under weak alkaline conditions and formed a layer of mesoporous ZrO_2_ on the surface of the SiO_2_ nanoparticles. When the reaction was completed, SiO_2_@ZrO_2_ NSPs were obtained by centrifuging (10000 r/min) the reaction solution. In order to obtain SiO_2_@ZrO_2_ NSPs with a cavity, we dissolved the solid SiO_2_@ZrO_2_ NSPs in 120 mL of deionized water (dH_2_O). Then, 1 mL of 1 M NaOH solution was added and stirred for 4 h at 80 °C. The mixed solution was centrifuged and washed for 3 times with dH_2_O to obtain the SiO_2_@ZrO_2_ NSPs. After that, we used the surface area and porosimetry analyzer to measure the specific surface area and pore diameter of the SiO_2_@ZrO_2_ NSPs.

#### Synthesis of CuO-SiO_2_@ZrO_2_ NSPs

In the experiment, we synthesized the CuO-SiO_2_@ZrO_2_ NSPs based on SiO_2_@ZrO_2_ NSPs. The detailed process is as follows. 40 mg of SiO_2_@ZrO_2_ NSPs were dissolved in 3 mL of dH_2_O. Then, 0.5 g of CuSO_4_·5H_2_O was added into the solution and ultrasonically dispersed. The solvent of the mixed solution was then drained by a vacuum pump, so that CuSO_4_·5H_2_O was entered the cavity of the SiO_2_@ZrO_2_ NPs. After that, dissolve the solid powder with 25 mL of absolute ethanol., and the solution was adjusted to alkaline system by adding 2 mL of ammonia (pH>9) 53,54. The mixed solution was refluxed for 2.5 h at 75 °C. When the reaction was completed, it was centrifuged and washed 3 times with dH_2_O. Finally, the precipitates were treated with HCl (0.20%) to remove the free CuO nanoparticles in the outside shell of the SiO_2_@ZrO_2_ NSPs, thus the CuO-SiO_2_@ZrO_2_ NSPs were obtained. Then, we used X-ray diffraction (XRD) test to verify that CuO nanoparticles were successfully loaded into SiO_2_@ZrO_2_ NSPs.

#### Preparation of the IQuCS@Zr NSPs

20 mg of the CuO-SiO_2_@ZrO_2_ NPs, 20 mg of Qu, 3 mL of 1,4-dioxane, 1.5 mL of IL, 5 mL of absolute ethanol and 5 mL of dH_2_O were added to a glass conical flask (25 mL) and dispersed evenly by ultrasonic bath. Then the mixed solution was pumped into a viscous liquid using a vacuum pump. Finally, the precipitates were centrifuged and washed for 3 times with dH_2_O to obtain the IQuCS@Zr NPs. After that, we used the surface area and porosimetry analyzer to measure the specific surface area and pore diameter of the IQuCS@Zr NPs. We also used the thermogravimetric analysis to determine the contents of Qu and IL loaded on SiO_2_@ZrO_2_ NPs.

#### Preparation of the IQuCS@Zr-PEG NSPs

mPEG-SH can be used to modify a pharmaceutical carrier to improve the biocompatibility of the carrier. In the experiment, mPEG-SH was used to improve the biocompatibility of the IQuCS@Zr NSPs. 20 mg of the as-prepared IQuCS@Zr NSPs, 10 mg of mPEG-SH and 15 mL of Tris-HCl bufter (pH=8.5) were added into 25 mL of erlenmeyer flask and dispersed evenly by ultrasonic bath. After stirring for 8 h at room temperature, the mixed solution were centrifuged and washed for 3 times with PBS (pH=7.4) to obtain the IQuCS@Zr-PEG NSPs.

### Determination of dissolved oxygen generated by CuO-SiO_2_@ZrO_2_ NSPs under microwave irradiation

In the experiment, the dissolved oxygen reagents were used to qualitatively determine the oxygen generation effect of the CuO-SiO_2_@ZrO_2_ NSPs. In the qualitative testing process, we divided the experiment into 6 groups: the dH_2_O group and the PBS group (the control groups); the CuO-SiO_2_@ZrO_2_ NSPs+dH_2_O group and the CuO-SiO_2_@ZrO_2_ NSPs+PBS group (the no MW irradiation groups); the MW+CuO-SiO_2_@ZrO_2_+dH_2_O NSPs group and the MW+CuO-SiO_2_@ZrO_2_+PBS NSPs group (the MW irradiation oxygen generation groups). During the test, the concentration of the CuO-SiO_2_@ZrO_2_ NSPs solution was 5 mg/mL, PBS was used as solvent (pH=5.5), the power of MW (450 MHz) irradiation was 0.6 W and time was 5 min. After MW irradiation, two drops of dissolved oxygen reagent I and dissolved oxygen reagent II were added to the test tube respectively. After the static reaction proceeded for 5 mins, a few droplets of dissolved oxygen reagent III were added. This can detect the oxygen generation capacity of CuO-SiO_2_@ZrO_2_ NSPs.

In this experiment, the microcomputer DO-BOD detector was used to quantitatively measure the oxygen generation effect of the CuO-SiO_2_@ZrO_2_ NSPs under MW irradiation. We divided the experiment into 4 groups: the PBS (pH=5.5) +MW group, the dH_2_O+MW group, the CuO-SiO_2_@ZrO_2_ NSPs+MW+dH_2_O group, and the CuO-SiO_2_@ZrO_2_NSPs+MW+PBS (pH=5.5) group, wherein the concentration of the CuO-SiO_2_@ZrO_2_ NSPs solution was 5 mg/mL. After 5 min of MW irradiation, the concentration of oxygen in the solution was quantitatively determined by the DO-BOD.

### Cellular immunofluorescence experiment

In this work, immunofluorescence assay was used to verify the IQuCS@Zr-PEG NSPs downregulate the expression of HIF-1α under MW irradiation. We divided the experiment into 4 groups. The control group, the MW+25 μg/mL IQuCS@Zr-PEG NSPs group, the MW+50 μg/mL IQuCS@Zr-PEG NSPs group, the MW+100 μg/mL IQuCS@Zr-PEG NSPs group. Human lung adenocarcinoma A549 cells were first laid in 6-well plate and cultured. Then the cells were treated by 200 μM/L CoCl_2_ solution to establish hypoxic state. After 24 h, the solution was removed, and the corresponding concentration of the IQuCS@Zr-PEG NSPs were added to the 6 well plates with incubation continued for 24 h. The cells were irradiated by MW with 1 min (2 W). After 24 h of incubation, the cells were digested for immunofluorescence staining by using anti-HIF-1α antibody. The staining process mainly included the following steps: (1) the cells were fixed at 4 °C overnight. (2) Washed off the fixing solution with washing solution. (3) Added the blocking solution for 1h. (4) Aspirated the blocking solution and add the primary antibody. (5) Added the secondary antibody after washing with the washing solution. After that, immunofluorescence staining and photography (Olympus X71, Japan) were performed.

### ^99m^Tc-HL91 labeled reoxygenation imaging experiment

In this study, ^99m^Tc-HL91 tracer labeled imaging was used to evaluate the reoxygenation in BALB/c nude mice tumors. We randomly divided BALB/c nude mice bearing the A549 cells into 5 groups (3 mice in one group): The control group (the group without any treatment, group one), the Qu group (group two), the IQuCS@Zr-PEG NSPs group (the group with experimental materials but without MW irradiation, group three), the IL-Quercetin-CuO-SiO_2_@ZrO_2_-PEG nanosuperparticles (IQuS@Zr-PEG NSPs)+MW group (the group without CuO to generate oxygen, group four), the IQuCS@Zr-PEG NSPs+MW group (the experimental oxygen generation group, group five). The experimental materials were injected into the body from the tail vein of the mice (concentration was 50 mg/kg). At 4 h, 0.1 mCi of 4, 9-diazo-3, 3, 10, 10-tetramethyldodecane-2, 11-dione oxime (^99m^Tc-HL91) tracer via tail vein of mice. At 6 h, BALB/c nude mice in the fourth and fifth groups were treated with MW. Finally, BALB/c nude mice were immobilized at a distance of 5 cm from the probe of SPECT imager. The reoxygenation status in the tumor microenvironment was obtained by scanning the tumor area with small animal SPECT scanning.

### Microwave heating effect of the IQuCS@Zr-PEG NSPs *in vitro*

We evaluated the MW sensitivity of the IQuCS@Zr-PEG NSPs by MW heating *in vitro*. The IQuCS@Zr-PEG NSPs were dissolved into saline solution to obtain the different concentrations (0, 2.5, 5, 7.5, 10 mg/mL) solution. The power of MW was 0.6 W and time was 5 min. Saline solution (0 mg/mL) was set as the blank control group. In the MW irradiation process, we used the FLIR system to monitor the temperature change of the solution in real-time and record the temperature every 10 s.

### Cytotoxicity test

We used the MTT assay to validate the cytotoxicity of the IQuCS@Zr-PEG NSPs. Human lung adenocarcinoma A549 cells were cultured in 96-well plate for 24 h (5% CO_2_, 37 °C). The IQuCS@Zr-PEG NPs with different concentration (0, 6.25, 12.5, 25, 50, 100, 200 μg/mL) were then added and co-cultured with the human lung adenocarcinoma A549 cells. After 24 h, the MTT solution was added and incubated with the cells for 4 h. After removing all the solvent in the 96-well plates, 200 μL of dimethyl sulfoxide (DMSO) was added into each well. Finally, the absorbance at 490 nm in each of the 96-well plates was measured using the enzyme labelling apparatus.

### Inhibition of tumor cells *in vitro*

MTT assay were used to test the inhibitory effect of the IQuCS@Zr-PEG NSPs on human lung adenocarcinoma A549 cells. First, human lung adenocarcinoma A549 cells were cultured in 7 well plates for 24 h (37 °C, 5% CO_2_) to achieve the experimental requirements. We divided the cell experiments into 7 groups which consisted of the blank control group; the MW+RT group; the RT+MW+IQuCS@Zr-PEG NSPs (0 μg/mL) group; the RT+MW+IQuCS@Zr-PEG NSPs (25 μg/mL) group; the RT+MW+IQuCS@Zr-PEG NSPs (50 μg/mL) group; the RT+MW+IQuCS@Zr-PEG NSPs (100 μg/mL) group; and the RT+MW+IQuCS@Zr-PEG NSPs (200 μg/mL) group. During the experiment, the IQuCS@Zr-PEG NSPs contained the same amount of Qu (26.17%), the MW power was performed by 0.6 W and time was 5 min. After MW irradiation, the A549 cells were incubated in 96 well plates for 48 h and 24 h, respectively. Finally, the cell viability and cell inhibition were measured by MTT assay.

### Acute toxicity test of animals

We verified the effects of the IQuCS@Zr-PEG NSPs on the normal mice through acute toxicity experiments in animals. Healthy mice were divided into five groups, three of each group. Then, different concentrations (0, 25, 50, 75, and 100 mg/kg) of the IQuCS@Zr-PEG NSPs were injected into the tail vein of the mice. During the experiment, the growth state of the mice was observed daily, and the body weight of the mice was recorded. After 15 days, the blood of the mice was collected for blood biochemical and blood routine analysis. Then, the mice were sacrificed and the main organs of the mice were removed including spleen, heart, lung, liver and kidney. Finally, formalin-fixed tissue was used for histological study.

### Combined treatment of microwave thermal therapy and radiation therapy experiment *in vivo*

In this experiment, we selected 16±1 g BALB/c nude mice bearing the human lung adenocarcinoma A549 cells as the experimental models. This model was used to assess the inhibitory effect of the IQuCS@Zr-PEG NSPs in human lung adenocarcinoma A549 cells. In the experiment, we randomly divided BALB/c nude mice bearing the A549 cells into 5 groups for treatment (4 mice in one group, the tumor volume was 150±10 mm^3^). The control group (the group without any treatment, group one); the Qu+RT group (the Qu sensitization RT group, group two); the IQuCS@Zr-PEG NSPs+RT group (the group with experimental materials and RT but without MW irradiation, group three); the IQuS@Zr-PEG NSPs+RT+MW group (the group without CuO to generate oxygen, but there was the radiosensitizer of Qu+MW+RT, group four); and the IQuCS@Zr-PEG NSPs+RT+MW group (the experimental oxygen generation group, group five). Among them, the power of MW and the MW time were 0.9 W and 5min respectively, the materials contained the same Quercetin content, the radiotherapy dose and time was also the same (8 Gy, 2 min). The treatment was started at 6 h after the tail vein injection of the materials, and used the FLIR system to monitor and recorded the temperature and thermal imaging results. The calculation method of tumor volume is D×d^2^/2 (D is the longest diameter of the tumor and d is the shortest diameter of the tumor). In the course of experiment, changes in tumor volume of each mouse were recorded every two days and photographs of the BALB/c nude mice were taken. BALB/c nude mice were sacrificed after 14 days. The major organ tumors of the BALB/c nude mice were fixed with 4% neutral formaldehyde.

### Experiment of detecting apoptosis by TUNEL staining

During the course of the experiment, we examined the tumor tissue of the mice using standard histological techniques. The deoxyuride-5-triphosphate biotin nick end labelling (TUNEL) staining was then used to assess the apoptotic effect of tumor cells. The TUNEL staining mainly included the following steps: making paraffin tumor tissue sections, removing paraffin, treating the tissue with Proteinase K working solution for cell permeation (20 min), adding TUNEL reaction solution for reaction (1 h), adding converter-POD reaction solution for reaction (30 min), color reaction with substrate DAB for 10 min, and finally the experimental sections were obtained by staining with Hematoxylin. Photographs were taken by using an optical microscope to obtain apoptosis results in the tumor tissue region.

### Immunohistochemistry experiments

We used the mouse hypoxia-inducible factor HIF-1 immunohistochemistry kit to assess the expression of HIF-1α in tumor tissue regions. The processes of immunohistochemical staining of HIF-1α factor were as follows: making paraffin tumor tissue sections, removing paraffin wax, performing gradient hydration, antigen retrieval, incubating with blocking buffer for 30 min (37 °C), adding ready-to-use primary antibody and incubating overnight at 4 °C, washing slices with TBS-T, adding the anti-HIF-1 aipha antibody diluted in antibody dilution and incubating for 30 min (37 °C), adding the Tween 20 and incubating for 20 min (37 °C), adding the HRP-SA and incubating for 30 min (37 °C), then washing the sections with TBS-T solution, staining with DAB chromogenic reagent to obtain immunohistochemical sections. Finally, photographs were taken by using an optical microscope (Olympus X71, Japan) to obtain the expression of HIF-1α in tumor tissues.

### CT Imaging experiment

To evaluate the CT imaging effect of CuO-SiO_2_@ZrO_2_ NSPs, we performed CT imaging experiments *in vitro* and* in vivo*. First, we dispersed different concentrations (1-10 mg/mL) of the CuO-SiO_2_@ZrO_2_ NSPs in a test tube. Then, we used the CT scanning imager to scan the sample in the CT imaging test tube and recorded the corresponding CT value. *In vivo* CT capability was tested by injecting 50 mg/kg of the IQuCS@Zr-PEG NSPs into the tail vein of mice. Finally, the mice were scanned with CT scanning imager at 0, 3, 6, 9, 24 h to obtain the corresponding *in vivo* CT values, thus obtaining the *in vivo* CT imaging effect of the IQuCS@Zr-PEG NSPs.

### Statistical Analysis

All experimental data in this research were expressed as mean ± standard deviation (S.D). Statistical analysis (*P<0.05, **P<0.01 and *** P<0.001).

## Results and Discussion

In typical synthesis, SiO_2_@ZrO_2_ NSPs with adjustable cavity size were prepared by using SiO_2_ nanoparticles as templates. CuO-SiO_2_@ZrO_2_ NSPs were synthesized by confining CuO nanoparticles into the cavity of SiO_2_@ZrO_2_ NSPs. Next, IL and Qu were introduced into the CuO-SiO_2_@ZrO_2_ NSPs by physical negative pressure to prepare the IQuCS@Zr NSPs. Finally, the IQuCS@Zr NSPs were surface modified by mPEG-SH to obtain the IQuCS@Zr-PEG NSPS with good biocompatibility.

### Characterization of the IQuCS@Zr-PEG NSPs

**Figure 1:**
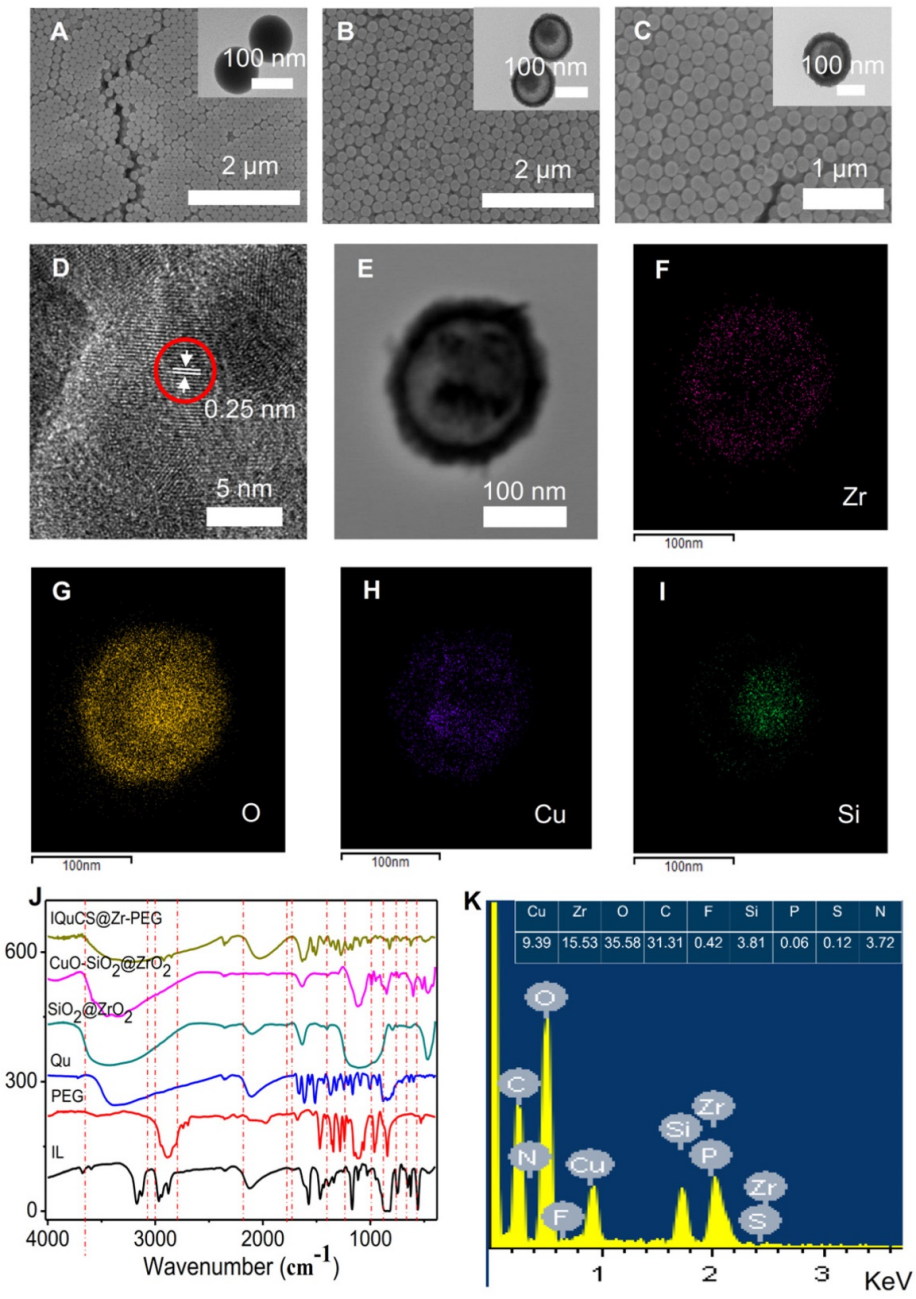
** Characterization of physical and chemical properties of the IQuCS@Zr-PEG NSPs.** (**A**) SiO_2_ nanoparticles. (**B**) SiO_2_@ZrO_2_ NSPs. (**C**) CuO-SiO_2_@ZrO_2_ NSPs. (**D**) Lattice picture of CuO nanoparticles in CuO-SiO_2_@ZrO_2_ NSPs. (**E**) Original image for taking a mapping of CuO-SiO_2_@ZrO_2_ NSPs. (**F**) Zr element. (**G**) O element. (**H**) Cu element. (**I**) Si element. (**J**) FT-IR spectrum of IQuCS@Zr-PEG NSPs. (**K**) The EDS diagram of the IQuCS@Zr-PEG NSPs.

Transmission electron microscopy (TEM) and scanning electron microscopy (SEM) were used to characterize the structure and morphology of SiO_2_ nanoparticles, SiO_2_@ZrO_2_ NSPs and CuO-SiO_2_@ZrO_2_ NSPs (**Figure 1A-C**). Figure 1A shows the solid structure of the SiO_2_ nanoparticles and the particle size is 133.4±12 nm (**Figure S1A**). As shown in the Figure 1B that the SiO_2_@ZrO_2_ NSPs display a mesoporous sandwich structure and the particle size is 173.71±15 nm (**Figure S1B**). The mesoporous sandwich structure of SiO_2_@ZrO_2_ NSPs can be used as a carrier for carrying CuO nanoparticles and drugs of Qu, *etc*. As shown in Figure 1C, we can clearly see that the CuO nanoparticles are loaded into the cavity of the SiO_2_@ZrO_2_ NSPs and the particle size of CuO-SiO_2_@ZrO_2_ NSPs is 174.52±12 nm (**Figure S1C**). We used a surface area and porosity analyzer to measure the specific surface area and pore size of SiO_2_@ZrO_2_ NSPs (**Figure S2A, B**). The nanosuperparticles has a high specific surface area 296.10 m^2^/g and a pore size of 4.0 nm.

High resolution TEM imaging (HRTEM) was used to verify that the CuO nanoparticles were encapsulated in SiO_2_@ZrO_2_ NSPs. It is shown from the HRTEM diagram (**Figure 1D**) that the CuO nanoparticles have high crystallinity and a lattice spacing of 0.25 nm. **Figure 1E** is the original image for taking a mapping of CuO-SiO_2_@ZrO_2_ NSPs. The elemental mapping of the CuO-SiO_2_@ZrO_2_ NSPs demonstrates the homogeneous distribution of Zr, O Cu and Si in the NSPs (**Figure 1F-I**). From this we can see clearly and intuitively that CuO nanoparticles are confined in the SiO_2_@ZrO_2_ NSPs. We used the XRD to further verify the Synthesis of CuO-SiO_2_@ZrO_2_ NSPs (**Figure S2C**). From Figure S2C, we can see that there are no obvious peaks in mesoporous sandwich SiO_2_@ZrO_2_ nanosuperparticles, indicating the amorphous nature. XRD pattern of CuO-SiO_2_@ZrO_2_ nanosuperparticle shows many peaks, especially two sharp peaks at 35.57° and 38.67°. The XRD peaks of CuO-SiO_2_@ZrO_2_ match well with the standard profile 55-57. In order to verify the presence of IL, Qu and modification of PEG, we used the Fourier transform infrared spectroscopy (FTIR) to study the functional groups of IQuCS@Zr-PEG NSPs (**Figure 1J**). The vibration peaks at 3452 cm^-1^, 1051 cm^-1^ can be assigned to C-O and O-H stretching, respectively. The above-mentioned characteristic peaks illustrate that Qu and PEG can be introduced into CuO-SiO_2_@ZrO_2_ NSPs 58,59. In addition, typical absorption peak of P-F at 851 cm^-1^, we can easily distinguish the tensile peak at 1172 cm^-1^ and the vibration peak at 1471 cm^-1^ and 1577 cm^-1^ of the imidazole ring, confirming that IL is encapsulated into the IQuCS@Zr-PEG NSPs 60,61. We used Energy-dispersive X-ray spectra (EDS) to verify the feature elements in IQuCS@Zr-PEG NSPs. As shown in **Figure 1K**, we can find that the relevant feature elements exist in EDS, which also proves that CuO nanoparticles, IL, Qu and PEG are successfully loaded into IQuCS@Zr-PEG SPCs. Among them, the content of Cu and Zr elements are 9.39% and 15.53%, respectively. We also used the surface area and porosity analyzer to measure the specific surface area and pore size of IQuCS@Zr (**Figure S2D,E**). The specific surface area of the IQuCS@Zr is 182.90 m^2^/g and the pore size of 2.2 nm. The mass loadings of IL and Qu within the IQuCS@Zr are determined to be 7.24% and 12.07% by TGA (**Figure S2F**), respectively. The successful synthesis of the IQuCS@Zr-PEG NSPs was verified by the above test methods.

### Oxygen generated by CuO-SiO_2_@ZrO_2_ NSPs for tumor reoxygenation

**Figure 2:**
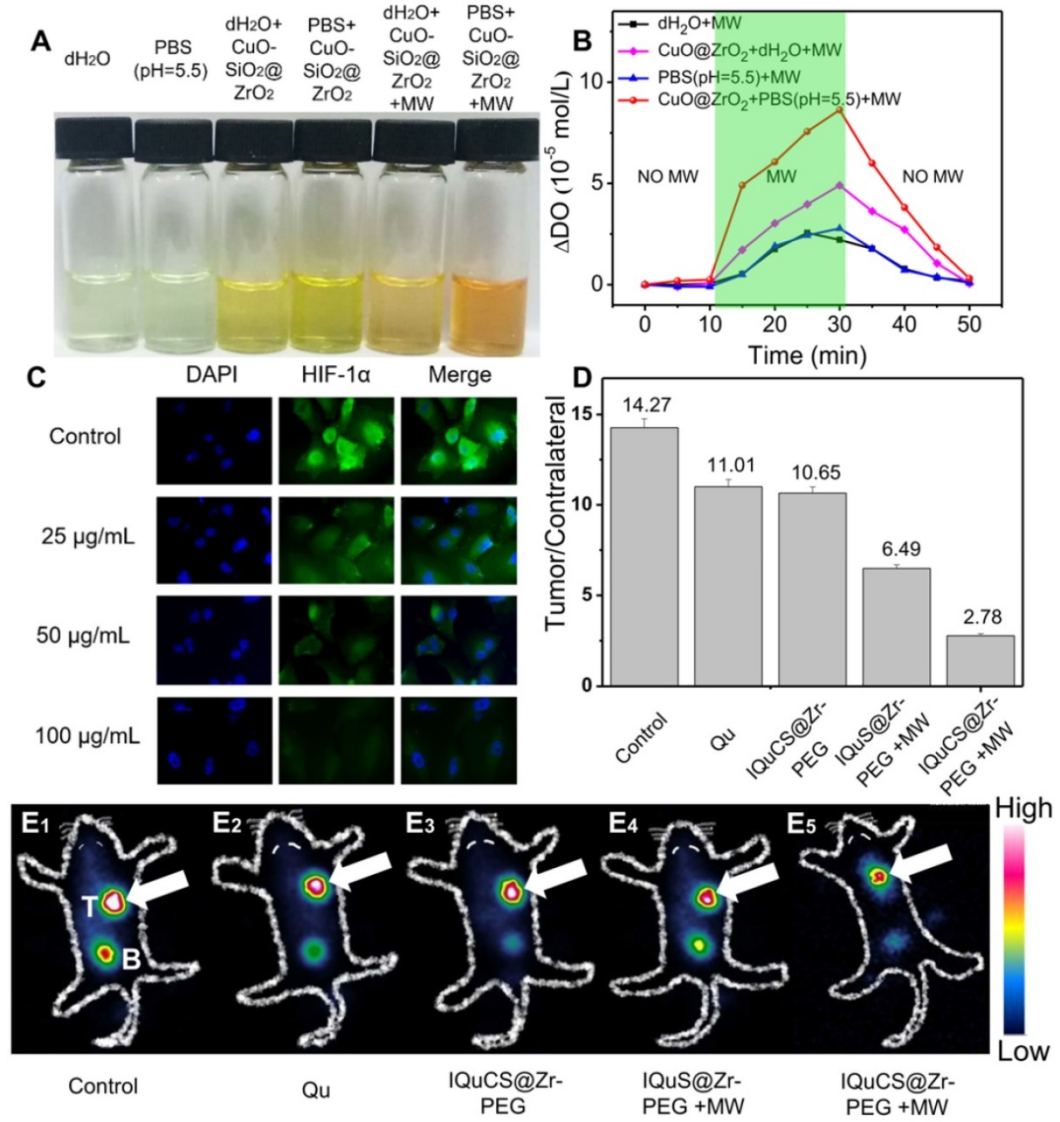
** Evaluation of the ability of CuO-SiO_2_@ZrO_2_ NSPs to generate oxygen, the expression of HIF-1α and ^99m^Tc-HL91 labeled reoxygenation imaging experiment.** (**A**) Picture of dissolved oxygen indicators for qualitative determination of oxygen production capacity of bare PBS (pH=5.5), dH_2_O, CuO-SiO_2_@ZrO_2_ NSPs+dH_2_O, CuO-SiO_2_@ZrO_2_ NSPs+PBS, CuO-SiO_2_@ZrO_2_ NSPs+MW+dH_2_O and CuO-SiO_2_@ZrO_2_ NSPs+MW+PBS. (**B**) The dissolved oxygen concentration of the solution was quantitatively determined by a microcomputer DO-BOD detector. (**C**) Immunofluorescence staining of nucleus and HIF-1α after treatment with the IQuCS@Zr-PEG NSPS ((DAPI (blue) and anti-HIF-1 aipha antibody (green)). (**D**) Quantitative analysis of the region of interest was performed using radioactive counting methods. (E_1_-E_5_) The small animal SPECT scanning detects the reoxygenation status of the different groups, T indicates tumor, B indicates bladder.

Hypoxic cells can seriously impede the treatment of RT. Therefore, tumor cells undergo reoxygenation during RT, which can significantly improve the therapeutic effect. In this paper, CuO nanoparticles are used to generate oxygen under MW irradiation to improve reoxygenation. We qualitatively determined the ability of CuO-SiO_2_@ZrO_2_ NSPs to generate oxygen by means of a dissolved oxygen indicator (**Figure 2A**). In this experiment, after adding the dissolved oxygen reagents, the color of the solution was deepened with the increase of dissolved oxygen. From **Figure 2A**, we can find that after adding the dissolved oxygen reagents to the blank group of PBS (pH=5.5) and dH_2_O, the color of the solution is almost transparent, indicating that there is negligible dissolved oxygen in the solution. As the CuO-SiO_2_@ZrO_2_ NSPs added into the solution, a weak increase of dissolved oxygen is seen. When the CuO-SiO_2_@ZrO_2_ NSPs were added with simultaneous MW irradiation, the large amount of dissolved oxygen increases remarkably in the solution. We used PBS as a solvent to quantitatively determine the dissolved oxygen generation of the CuO-SiO_2_@ZrO_2_ NSPs. Figure 2B is a quantitative determination of the concentration of oxygen generated by CuO-SiO_2_@ZrO_2_ NSPs under the irradiation by MW using a DO-BOD.

**Figure 2B** presents the quantitative evaluation of dissolved oxygen along with the duration of MW irradiation. Little dissolved oxygen (2.78 mol/L and 2.56 mol/L) is presented in bare dH_2_O and PBS under the irradiation by MW. The oxygen content shows a general rising trend with the increase of MW irradiation time. After MW treatment for 20 min, the dissolved oxygen generated CuO-SiO_2_@ZrO_2_ NSPs in bare dH_2_O is 4.91 mol/L. More significantly, CuO-SiO_2_@ZrO_2_ NSPs generated in bare PBS at pH=5.5 is 8.63 mol/L. It is calculated that the oxygen generated by the CuO-SiO_2_@ZrO_2_ NSPs under the irradiation by MW is 3.1 times than that of the bare PBS solvent. Therefore, once the CuO-SiO_2_@ZrO_2_ NSPs enter the tumor microenvironment, oxygen can be generated under MW irradiation, which upregulates tumor reoxygenation and enhances the treatment of hypoxic tumor.

### The downregulating of HIF-1α expression* in vitro*

In this study, the expression of HIF-1α was used to reflect the degree of hypoxia in tumor cells and the reoxygenation of tumor cells 62-64. As shown in **Figure 2C**, the human lung adenocarcinoma A549 cell nucleus and HIF-1α were stained with 2-(4-amidinophenyl)-6-indolecarbamidine dihydrochloride (DAPI, blue) and anti-HIF-1 aipha antibody (green). Compared with the control group, the immunofluorescence intensity decreased with the increase of the IQuCS@Zr-PEG NSPs concentration. This results indicate that the oxygen generated by the IQuCS@Zr-PEG NSPs under MW irradiation can effectively downregulate the expression of HIF-1α and improve the reoxygenation of tumor cells, thereby relieving the hypoxic state of tumor cells. It is also proved that when the IQuCS@Zr-PEG NSPs are indwelled in the tumor microenvironment, the oxygen can be continuously released under the irradiation by MW, which can effectively upregulate tumor reoxygenation and enhance the treatment of hypoxic tumor.

### Analysis of the results of ^99m^Tc-HL91 labeled reoxygenation imaging experiments

^99m^Tc-HL91 tracer was used to evaluate the reoxygenation status in BALB/c nude mice tumors (**Figure 2E_1_-2E_5_**) 65. The more ^99m^Tc-HL91 tracer is ingested in the tumor area, the brighter the color of the image will be, and the worse the degree of reoxygenation in the tumor cells. From **Figure 2E_1_**, we can find that the color of the control group is the brightest, and the results show that the degree of reoxygenation in the tumor cells of this group is the worst. The color of Qu (**Figure 2E_2_**) and IQuCS@Zr-PEG NSPs group (**Figure 2E_3_**) are still very bright, which indicate that the degree of reoxygenation in the tumor cells has not been significantly improved, so the therapeutic effect is poor. From the IQuS@Zr-PEG NSPs+MW group (**Figure 2E_4_**), we found that the color in the tumor area became lighter and the degree of reoxygenation improved, but the treatment efficiency was not obvious. From the imaging results of the experimental group in the tumor tissue area, we found that the oxygen generated by the IQuCS@Zr-PEG NSPs under MW irradiation can significantly improve the degree of reoxygenation of the tumor cells (**Figure 2E_5_**), which can be significantly enhanced the therapeutic efficiency. Moreover, we quantitatively analyzed the region of interest by radionuclide counting (**Figure 2D**). Among them, the Tumor/Contrlateral ratio refers to the count rate in the tumor tissue divided by the background count rate. From the figure we can find that the Tumor/Contrlateral ratios in the control group, Qu group, IQuCS@Zr-PEG NSPs group, IQuS@Zr-PEG NSPs+MW group and IQuCS@Zr-PEG NSPs+MW group is 14.27, 11.01, 10.65, 6.49 and 2.78, respectively. The smaller the Tumor/Contralateral ratio, the better the degree of reoxygenation. Therefore, when the IQuCS@Zr-PEG NSPs are enriched in the tumor microenvironment, it can release a large amount of oxygen under MW irradiation, which can significantly improve the reoxygenation state in tumor cells and enhance the combined therapeutic effect of RT and MWTT.

### Biocompatibility evaluation of the IQuCS@Zr-PEG NSPs *in vitro* and *in vivo*

**Figure 3:**
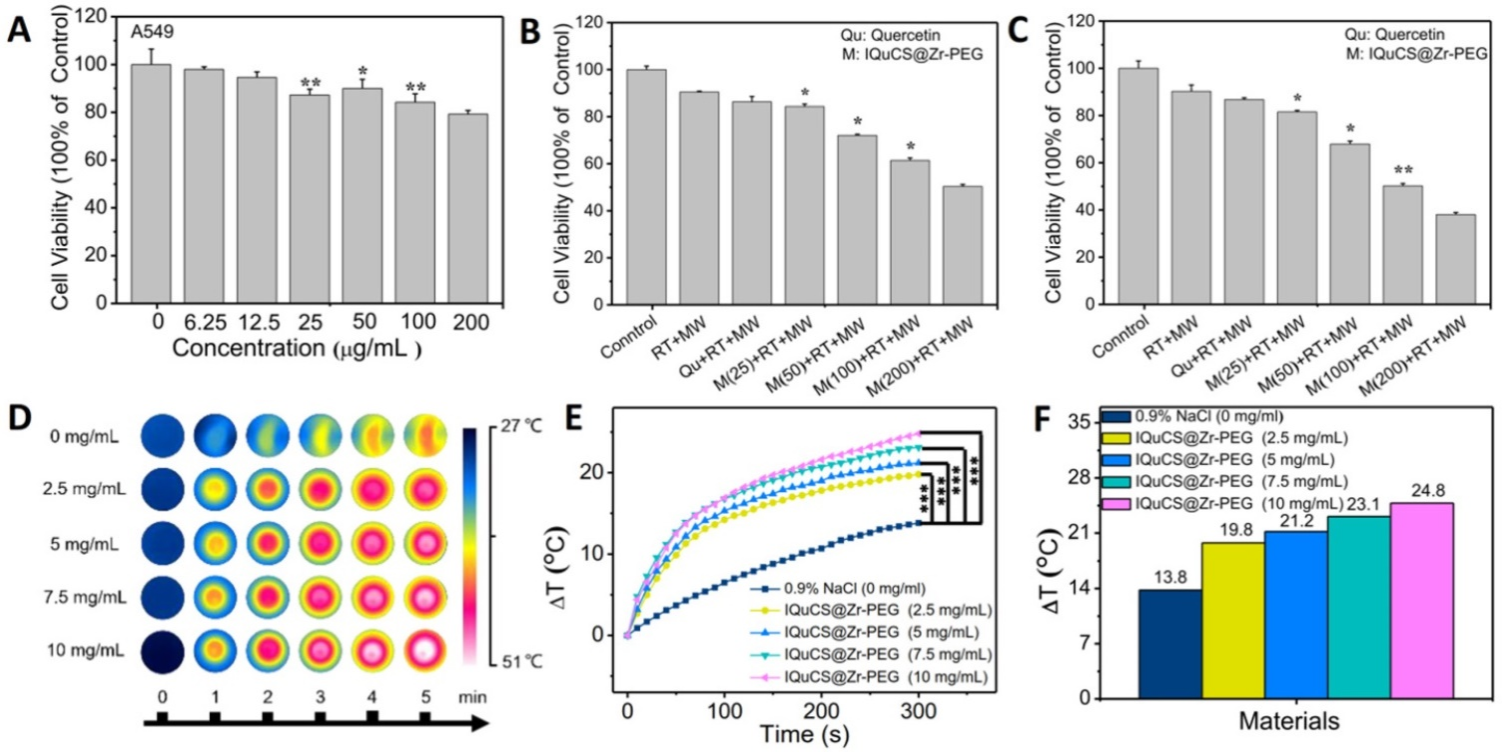
** The cell experiment and *in vitro* MW heating experiment results of the IQuCS@Zr-PEG NSPs**. (**A**) The viability of human lung adenocarcinoma A549 cells co-incubated with the IQuCS@Zr-PEG NSPs at different concentrations were determined by MTT assay (n=5). (**B**) The viability of human lung adenocarcinoma A549 cells under different treatments or different concentrations of IQuCS@Zr-PEG NSPs for 24 h (n=5). (**C**) The viability of human lung adenocarcinoma A549 cells under different treatments or different concentrations of IQuCS@Zr-PEG NSPs for 48 h (n=5). (**D**) Corresponding to (E) image of the FLIR thermal image. (**E**) Temperature-raising of different concentration of the IQuCS@Zr-PEG NSPs saline solution under the irradiation by MW. (**F**) The highest temperature rise chart was corresponding to (E). Analysis of statistical (*indicates P < 0.05, ** indicates P < 0.01 and *** indicates P < 0.001).

Human lung adenocarcinoma A549 cells were used to assess the effect of the IQuCS@Zr-PEG NSPs in inhibiting tumor cells *in vitro* 66-68. First, we explored the biological toxicity of the IQuCS@Zr-PEG NSPs. Even the concentration of the IQuCS@Zr-PEG NSPs reaches to 200 μg/mL, the cell viability is still 79.24% (**Figure 3A**), indicating that the low toxicity of the IQuCS@Zr-PEG NSPs. Furthermore, we have verified the side effects of the IQuCS@Zr-PEG NSPs on mice through acute toxicity experiments. The low toxicity of the IQuCS@Zr-PEG NSPs *in vivo* was demonstrated by 14-day weight changes test (**Figure S3A**), serum biochemical analysis (**Figure S3B**), routine blood examination (**Figure S3C**) and H&E staining test of the main organs (**Figure S3D**). **Figure 3B and 3C** show the inhibitory effect of the IQuCS@Zr-PEG NSPs on human lung adenocarcinoma A549 cells. The cell viability decreases with the increase of the concentration of the IQuCS@Zr-PEG NSPs under the same treatment (same Qu content, same RT dose and time, same MW irradiation time and power, same incubation time). These results indicate that the inhibitory effect of the IQuCS@Zr-PEG NSPs on human lung adenocarcinoma A549 cells are improved with increased IQuCS@Zr-PEG NSPs concentrations. The reason for this result is attributed to the increase in oxygen generated by CuO nanoparticles after MW irradiation as the increase of the IQuCS@Zr-PEG NSPs concentration, which upregulates tumor reoxygenation and improves the inhibitory effect on tumor cells.

### Evaluation of microwaveheating performance *in vitro*

The IQuCS@Zr-PEG NSPs present the effect of MW sensitization which is attributed to the confinement effect of IL in enclosed space 69. During the experiment, the temperature changes of the IQuCS@Zr-PEG NSPs was used the FLIR system to monitor in real-time under the irradiation by MW (frequency of 450 MHz). The MW duration of each material is 5 min and the power of MW is 0.6 W. **Figure 3D** is a thermal imaging image obtained by taking a screenshot every 1 min after real-time monitoring by FLIR. The figure shows that the color becomes more vivid with the concentration of the IQuCS@Zr-PEG NSPs increases, reflecting enhanced heating effect. **Figure 3E** demonstrates the temperature value curves of IQuCS@Zr-PEG NSPs with different concentrations in saline solution under MW irradiation. As shown in **Figure 3F**, the temperature of the control group (saline solution) increases to 13.8 °C during 5 min of MW irradiation. With the concentration of IQuCS@Zr-PEG NSPs increases, the temperature of the solution shows an overall upward trend. When the concentration is 10 mg/mL, the temperature of the saline solution increased to 24.8 °C, which is 11 °C higher than the saline solution in the control group. After 5 min of MW irradiation, the temperature of the IQuCS@Zr-PEG NSPs saline solution with 2.5, 5, 7.5 and 10 mg/mL increases to 19.8 °C, 21.2 °C, 23.1 °C and 24.8 °C, respectively. Moreover, compared with the control saline solution, the temperature changes of the IQuCS@Zr-PEG NSPs saline solution with 2.5, 5, 7.5 and 10 mg/mL is 6 °C, 7.4 °C, 9.3 °C and 11 °C. These results indicate that IQuCS@Zr-PEG NSPs saline solution has a very good heating effect under MW irradiation. It can be suitable for MWTT of tumor *in vivo*.

### Evaluation of combined tumor treatment effects of RT and MWTT *in vivo*

**Figure 4:**
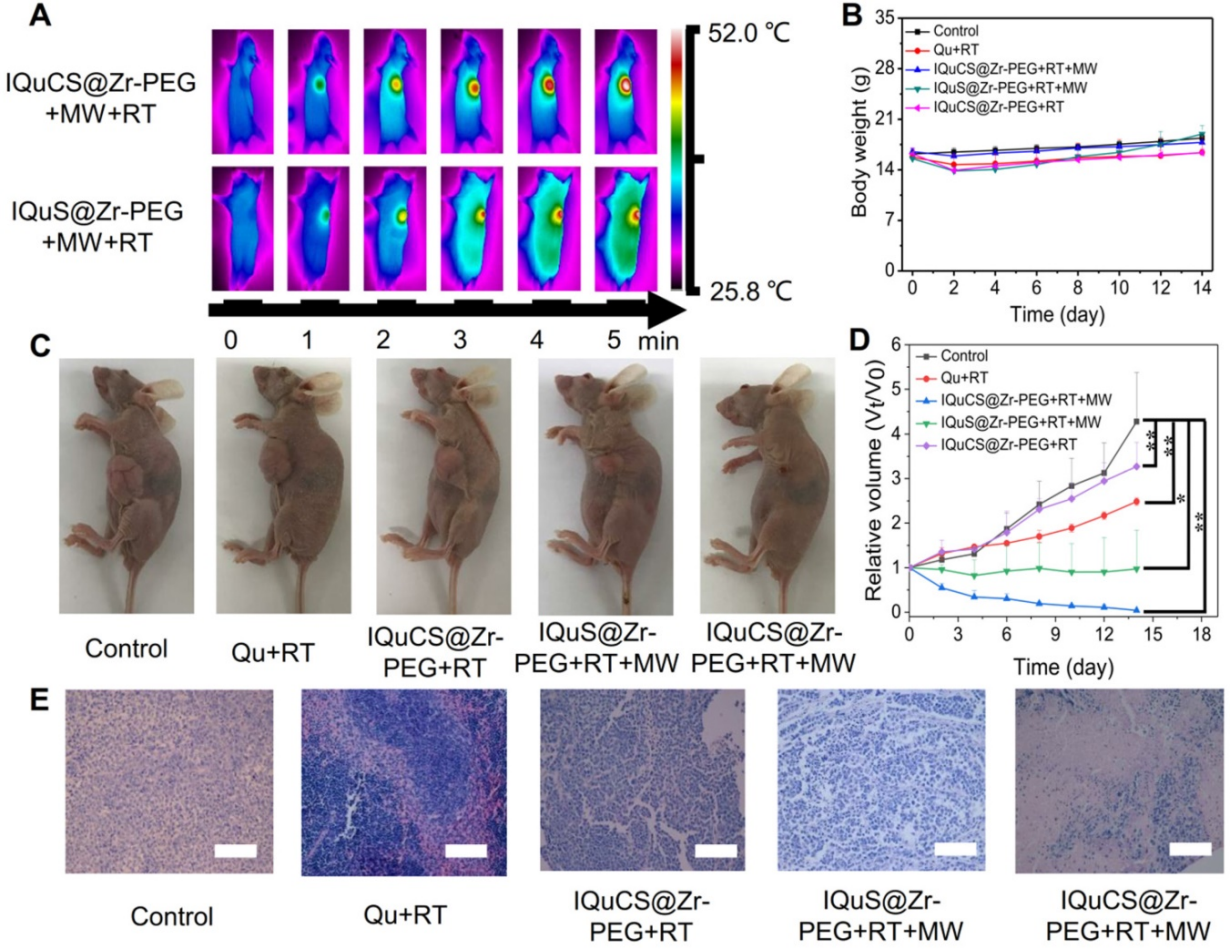
** Evaluation of *in vivo* treatment experiments of the IQuCS@Zr-PEG NSPs.** (**A**) FLIR map of mice in the IQuCS@Zr-PEG+RT+MW group and the IQuS@Zr-PEG+RT+MW group after MW irradiation per minute. (**B**) Body weight changes every two days during the 14 days of treatment in each mouse. (**C**) After 14 days, the mice were sacrificed, and the residual condition of the tumor after treatment in each group of mice. (**D**) The relative tumor volumes (Vt/V0) of each mouse within 14 days after treatment. (**E**) H&E stained sections of the tumor tissues were treated for 14 days after treatment in each group. The scale bar is 50 µm. Analysis of statistical (n=4, * indicates P < 0.05, ** indicates P < 0.01 and *** indicates P < 0.001).

According to the results of the appeal experiment, the IQuCS@Zr-PEG NSPs can continuously generate oxygen under MW irradiation, which can downregulate the expression of HIF-1α and upregulate tumor reoxygenation. Moreover, it has a good inhibiting and heating effect on tumor cells. We validated the antitumor effect of the IQuCS@Zr-PEG NSPs in BALB/c nude mice (**Scheme 1B**). Before the in vivo antitumor experiment, we study the biological distribution of IQuS@Zr-PEG NSPs in mice after intravenous injection. According to the biodistribution and tumor accumulation result (**Figure S4A**), within 24 h of injection, most of IQuS@Zr-PEG NSPs were concentrated in liver and spleen. The amount of IQuS@Zr-PEG NSPs at tumor site reached its highest value at 6 h, and then gradually decreased at 12 and 24 h slightly, indicating that the appropriate treatment time should be 6 h after injection. **Figure S4B** is a blood circulation profile for IQuS@Zr-PEG NSPs. From this figure, we find the content of IQuS@Zr-PEG NSPs in blood decreased with time, indicating that IQuS@Zr-PEG NSPs gradually entered the main organs and tumors from the blood and participated in physiological metabolism. In the experiment, we inoculated human lung adenocarcinoma A549 cells in the abdomen of BALB/c nude mice to establish a tumor model. BALB/c nude mice were divided into five groups (4 in each group). The without any treatment group (group one); the Qu+RT group (group two); the IQuCS@Zr-PEG NSPs+RT group (group three); the IQuS@Zr-PEG NSPs+RT+MW group (group four); and the IQuCS@Zr-PEG NSPs+RT+MW group (group five). Among them, group one was the control group, group two was the Qu sensitization RT group, group three was the experimental materials without MW irradiation+RT group, group four was the combined treatment of RT and MWTT, group five was the oxygen generation enhanced combined treatment of RT and MWTT (experimental group). **Figure 4A** is a FLIR thermal image of the IQuCS@Zr-PEG NSPs+RT+MW group and the IQuS@Zr-PEG NSPs+RT+MW group. As shown in the figure, we find that the two groups of mice have the same elevated temperature (about 52 °C) at the same time of MW in tumor site, so that the results of different treatment effects caused by different temperature rises can be excluded (**Figure S5A**). From **Figure 4B**, there is was no obvious changes in each group of mice of their body weight. The results indicate that the good biocompatibility of the IQuCS@Zr-PEG NSPs in mice.** Figure 4C** demonstrates the residual state of the tumor after each treatment of 14 mice in each group. From **Figure 4C**, compared with the other four groups, the IQuCS@sr-PEG NSPs+RT+MW group has the most significant treatment effect (Figure S5B). **Figure 4D** is a graph showing changes in tumor volume within 14 days after treatment in each experimental mouse. Compared with the IQuS@Zr-PEG NSPs+RT+MW group, the IQuCS@Zr-PEG NSPs+RT+MW group has a better treatment effect. This result indicates that the presence of CuO nanoparticles leads to an increase in tumor inhibition. It is proved that the effect of combined therapy of RT and MWTT is effectively improved by upregulateing tumor reoxygenation and reducing the hypoxia-induced treatment resistance through the oxygen generation. **Figure 4E** is the H&E stained tumor tissue section of each group of mouse tumor tissues. From the figure we can see that the cells in the control group are full and not destroyed. The cells in the IQuS@Zr-PEG NSPs+RT+MW group, the Qu+RT group and the IQuCS@Zr-PEG NSPs+RT group have been destroyed to different degrees, but the damage is not serious, which is the main cause of tumor recurrence. From the tumor tissue section of the experimental group, we can find that the tumor cells are severely damaged, so the treatment effect is most obvious. We analyze the H&E staining tissue sections results of the main organs of the mice and find that the IQuCS@Zr-PEG NSPs demonstrate no significant damage to each organ (**Figure S5C**). The results of antitumor experiments *in vivo* demonstrated that IQuCS@Zr-PEG NSPs can generate sufficient oxygen to upregulate tumor reoxygenation under the irradiation by MW, which can improve the combined treatment of RT and MWTT.

### Analysis of the results of TUNEL and immunohistochemistry

**Figure 5:**
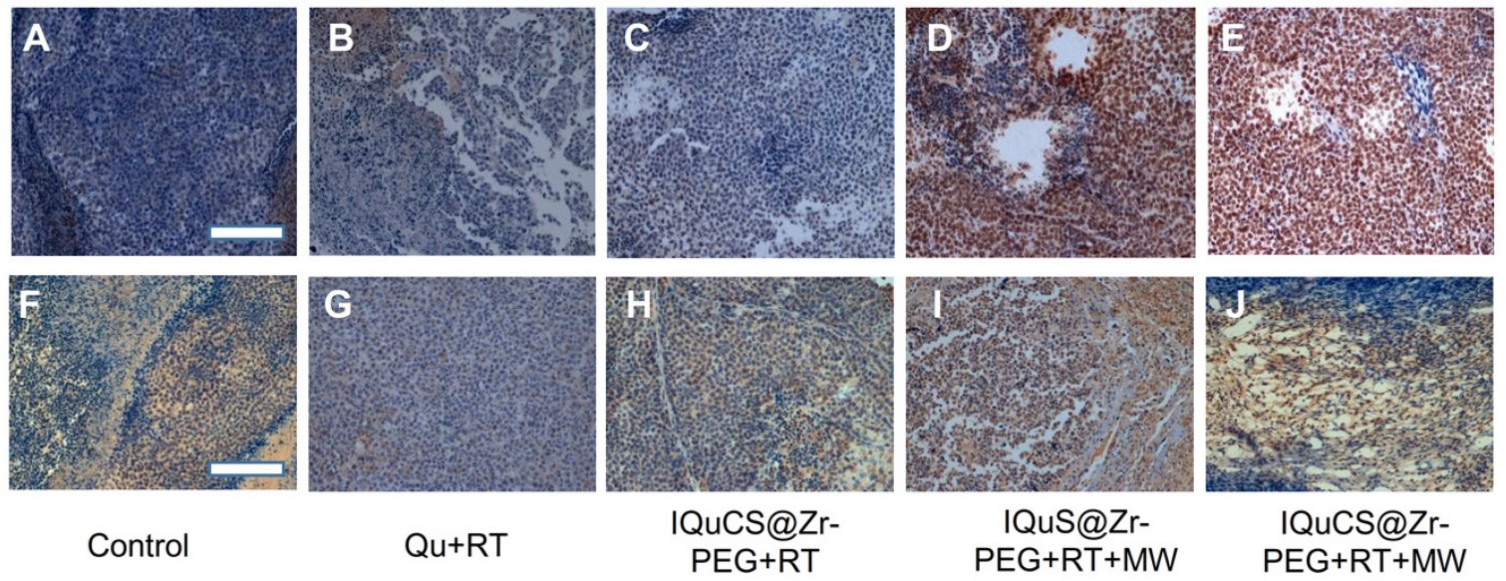
** Experimental results of TUNEL and immunohistochemistry.** (**A-E**) Figures A to E were the TUNEL results of the tumor tissues in the control group, Qu+RT group, IQuCS@Zr-PEG+RT group, IQuS@Zr-PEG+RT+MW group, and IQuCS@Zr-PEG+RT+MW group, respectively (magnification 200×). (**F-J**) Figures F to J were the immunohistochemistry results of the tumor tissues in the control group, Qu+RT group, IQuCS@Zr-PEG+RT group, IQuS@Zr-PEG+RT+MW group, and IQuCS@Zr-PEG+RT+MW group, respectively (magnification 200×). The scale bar is 50 µm.

In the experiment, TUNEL staining were used to evaluate the effect of apoptosis in tumor areas (**Figure 5A-E & Figure S6A-6E**) 70. TUNEL staining showed that almost no positive cells were found in the tumor cells in the control group (**Figure 5A & Figure S6A**). Fewer positive cells were found in the tumor cells of the Qu+RT group (**Figure 5B &Figure S6B**) and the IQuCS@Zr-PEG NSPs+RT group (**Figure 5C & Figure S6C**). Many positive cells were found in the tumor cells of the IQuS@Zr-PEG NSPs+RT+MW group (**Figure 5D & Figure S6D**). In the experimental group, we can see the distribution of a large number of positive cells (**Figure 5E & Figure S6E**). The surface of these positive cells was yellow-brown particles, the cytoplasm atrophied, and the nucleus ruptured with the formation of apoptotic bodies. These results indicate that IQuCS@Zr-PEG NSPs generate oxygen under MW irradiation, which can effectively upregulate tumor reoxygenation, thereby enhancing the combined treatment of RT and MWTT. Moreover, from the figure we can find that the better the treatment effect, the more apoptosis of tumor cells.

Mouse hypoxia inducible factor (HIF-1) immunohistochemistry were used to assess the HIF-1α in tumor tissue (**Figure 5F-5J & Figure S6F-6J**) 71,72. During the detection process, HIF-1α factor was stained with mouse HIF-1 immunohistochemical reagent to form brown-yellow granules or brown-yellow substances between cells. Moreover, the more brown-yellow substances found between cells, the more serious the hypoxia in the tumor region. From **Figure 5F** and **Figure S6F**, we find a large amount of brown-yellow substance in the intercellular space of the control group, and the results show that the degree of hypoxia in the tumor area was very serious. There are also many brownish-yellow substances in the Qu+RT group (**Figure 5G & Figure S6G**) and the IQuCS@Zr-PEG NSPs+RT group (Figure 5H, and Figure S6H), we find the hypoxia of the tumor tissue is not reduced, resulting in poor treatment effects. From the IQuS@Zr-PEG NSPs+RT+MW group (**Figure 5I & Figure S6I**), we find that the degree of hypoxia in the tumor area is reduced and there is no large amount of brown-yellow substance. In the experimental group, we find very few brown-yellow substances in the tumor tissue area. The results demonstrate that the oxygen generated by MW irradiation of the IQuCS@Zr-PEG NSPs could increase the reoxygenation capacity of tumor cells (**Figure 5J & Figure S6J**), thus enhancing the combination treatment of RT and MWTT.

### Evaluation of CT imaging capabilities

**Figure 6:**
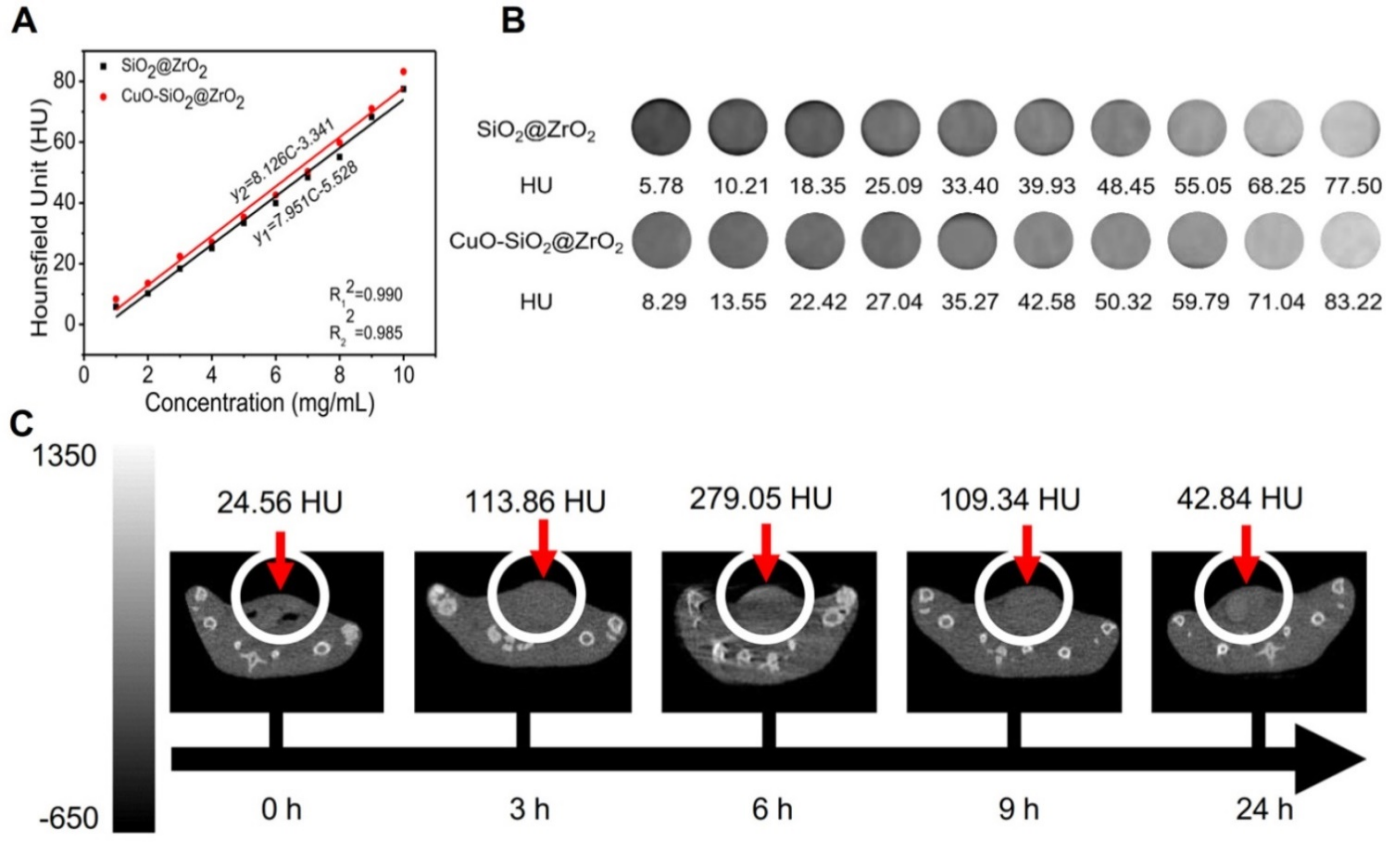
** The CT imaging effect of IQuCS@Zr-PEG NSPs *in vitro* and* in vivo*.** (**A,B**) The CT effect of different concentrations of SiO_2_@ZrO_2_ NSPs and CuO-SiO_2_@ZrO_2_ NSPs aqueous solutions *in vitro*. (**C**) After injection of 50 mg/kg of the IQuCS@Zr-PEG NSPs into tail vein of mice, the CT imaging results *in vivo* were measured at 0, 3, 6, 9 and 24 h, respectively.

Owing to the high atomic number and atomic mass of Cu and Zr, the as-obtained nanosuperparticles may be capable for enhancing the effect of CT imaging 73. We further explored the CT imaging capabilities of IQuCS@Zr-PEG NSPs. Firstly, we explored the effects of CT imaging capabilities of the SiO_2_@ZrO_2_ NSPs and CuO-SiO_2_@ZrO_2_ NSPs *in vitro*, and the concentrations of the materials ranged from 1 mg/mL to 10 mg/mL. From **Figure 6A and 6B** we can find that SiO_2_@ZrO_2_ NSPs and CuO-SiO_2_@ZrO_2_ NSPs have excellent CT imaging ability. As shown in the figure, in the same concentration of the solution, the imaging effect of CuO-SiO_2_@ZrO_2_ NSPs excels than SiO_2_@ZrO_2_ NSPs, which the main reason is that CuO nanoparticles are loaded.

Moreover, we can also find that within a certain concentration range, the HU values of CT increases as with concentration of the materials. **Figure 6C** shows the *in vivo* CT imaging results at 0, 3, 6, 9 and 24 h after intravenous injection of the IQuCS@Zr-PEG NSPs (50 mg/kg) in the tail vein of the experimental mouse, with HU values of 24.56, 113.86, 279.05, 109.34 and 42.84, respectively. From the date we can find that the IQuCS@Zr-PEG NSPs can gradually accumulate in the tumor tissue after entering the blood of mice, which peaks at 6 h and begins to fall in succession. Therefore, we chose 6 h after intravenous administration in tail vein of mice as the optimal treatment time. Through *in vivo* and *in vitro* CT experiments, we confirmed that the IQuCS@Zr-PEG NSPs have a very good CT imaging capabilities. We use the IQuCS@Zr-PEG NSPs for oncology treatment as well as visual monitoring of tumor treatment processes.

## Conclusion

In summary, we successfully prepared the promising multifunctional IQuCS@Zr-PEG NSPs which were based on CuO nanoparticles irradiated by MW to generate oxygen, thus upregulating tumor reoxygenation. The as-made IQuCS@Zr-PEG NSPs were prepared by templates method. Mesoporous sandwich SiO_2_@ZrO_2_ NSPs with adjustable cavity size were synthesized using SiO_2_ nanoparticles as templates. CuO-SiO_2_@ZrO_2_ NSPs with oxygen-generating ability under the irradiation by MW were prepared by confining CuO nanoparticles to the cavity of SiO_2_@ZrO_2_ NSPs. After that, the IL with perfect MW sensitivity effect was loaded into the CuO-SiO_2_@ZrO_2_ NSPs to enhance the treatment effect of MWTT. Radiosensitizer of Qu was introduced into the IL-CuO-SiO_2_@ZrO_2_ NSPs to obtain the IQuCS@Zr NSPs. Finally, we used mPEG-SH for surface chemical modification to obtain the IQuCS@Zr-PEG NSPs with good biocompatibility. Through the DO-BOD test, the IQuCS@Zr-PEG NSPs generated 3.10 times the oxygen concentration of bare solution (PBS) under MW irradiation. Therefore, when the IQuCS@Zr-PEG NSPs are indwelled the tumor microenvironment, they can continuously release oxygen under the irradiation by MW. Reoxygenation of hypoxic cells causes reshaping of the tumor microenvironment, thereby reducing the failure of RT due to hypoxia. Furthermore, the IQuCS@Zr-PEG NSPs contain the radiosensitizer of Qu and the MW sensitizer with the IL, which can significantly enhance the combined treatment of RT and MWTT. And through the analysis of cellular immunofluorescence and immunohistochemistry experiments, we found that the IQuCS@Zr-PEG NSPs can downregulate the expression of HIF-1α under MW irradiation, which reflected the reoxygenation of tumor cells from the side. ^99m^Tc-HL91 labeled reoxygenation imaging experiment demonstrated that IQuCS@Zr-PEG NSPs can generate oxygen under microwave irradiation, which significantly upregulated tumor reoxygenation. The results of cell experiments and antitumor experiments* in vivo* showed that the IQuCS@Zr-PEG NSPs had an excellent inhibitory effect on tumor cells, and the tumor inhibition rate reached 98.62%. Furthermore, owing to the high relative atomic number of Zr and Cu elements can enhance the CT imaging ability of the IQuCS@Zr-PEG NSPs, we can use it to monitor the cancer treatment process in real-time. To the best of our knowledge, IQuCS@Zr-PEG NSPs represent the first MW-responsive reoxygenation enhancer that efficiently reshapes tumor microenvironment, which is highly desired for versatile therapeutic efficacy.

## Supplementary Material

Supplementary figures.
