# Supplementary material for: Tumor reoxygenation for enhanced combination of radiation therapy and microwave thermal therapy using oxygen generation in situ by CuO nanosuperparticles under microwave irradiation
Source: Theranostics. 2020 Mar 25;10(10):4659–75. doi: 10.7150/thno.42818 (PMC7150478; doi:10.7150/thno.42818)
Supplement: Supplementary file 1 — Supplementary figures. [file thnov10p4659s1.pdf]

Supplementary Material

Tumor reoxygenation for enhanced combination of radiation therapy and microwave thermal therapy using oxygen generation in situ by CuO nanosuperparticles under microwave irridiation

Authors: Zengzhen Chen[1, 5], Wenna Guo[1, 6], Qiong Wu[1], Longfei Tan[1], Tengchuang Ma[2]*, Changhui Fu[1], Jie Yu[3], Xiangling Ren[1], Jianming Wang[4], Ping Liang[3]*, Xianwei Meng[1]*

Affiliations:

1. Laboratory of Controllable Preparation and Application of Nanomaterials, Key Laboratory of Cryogenics, Technical Institute of Physics and Chemistry, Chinese Academy of Sciences, No. 29 East Road Zhongguancun, Beijing 100190, People's Republic of China.

2. Department of Nuclear Medicine, Harbin Medical University Cancer Hospital, Harbin 150086, People's Republic of China.

3. Department of Interventional Ultrasound, Chinese PLA General Hospital, Beijing 100853, People's Republic of China.

4. Beijing Institute of Fashion Technology. No. A2, East Yinghua Street, Chaoyang District, Beijing 102209, People's Republic of China.

5. University of Chinese Academy of Sciences, Beijing 100049, People's Republic of China.

6. School of Information Engineering, Inner Mongolia University of Science and Technology, Baotou 014010, People's Republic of China.

Corresponding authors:

Tengchuang Ma (matengchuang1988@126.com), Ping Liang (liangping301@hotmail.com) and Xianwei Meng (mengxw@mail.ipc.ac.cn)

Figures in supplementary material

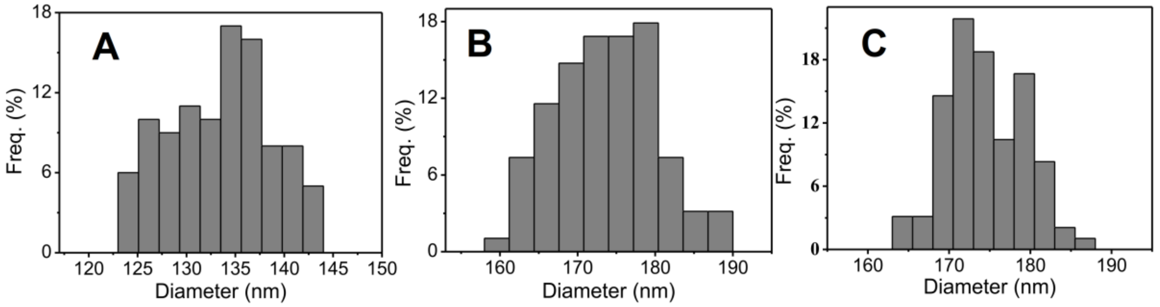

Figure S1. Characterization of the IQuCS@Zr-PEG NSPs. (A) Particle size distribution of SiO_2 nanoparticles. (B) Particle size distribution of SiO_2@ZrO_2 NSPs. (C) Particle size distribution of CuO-SiO_2@ZrO_2 NSPs.

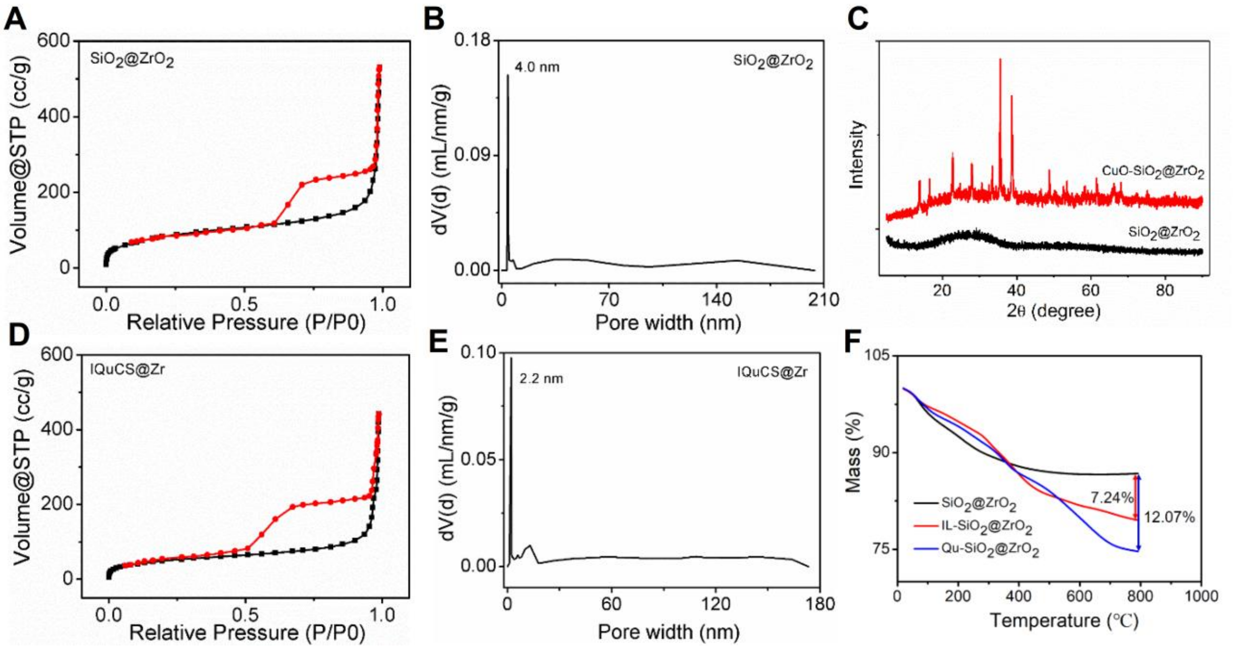

Figure S2. The specific surface area and pore diameter of the SiO_2@ZrO_2 NSPs and IQuCS@Zr NSPs, The XRD analysis of SiO_2@ZrO_2 NSPs and CuO-SiO_2@ZrO_2 NSPs. (A) The specific surface area of the SiO_2@ZrO_2 NSPs. (B) The pore diameter of the SiO_2@ZrO_2 NSPs. (C) The XRD analysis of SiO_2@ZrO_2 NSPs and CuO-SiO_2@ZrO_2 NSPs. (D) The specific surface area of the IQuCS@Zr NSPs. (E) The pore diameter of the IQuCS@Zr NSPs. (F) TGA results of SiO_2@ZrO_2 NSPs, IL-SiO_2@ZrO_2 NSPs and Qu-SiO_2@ZrO_2 NSPs.

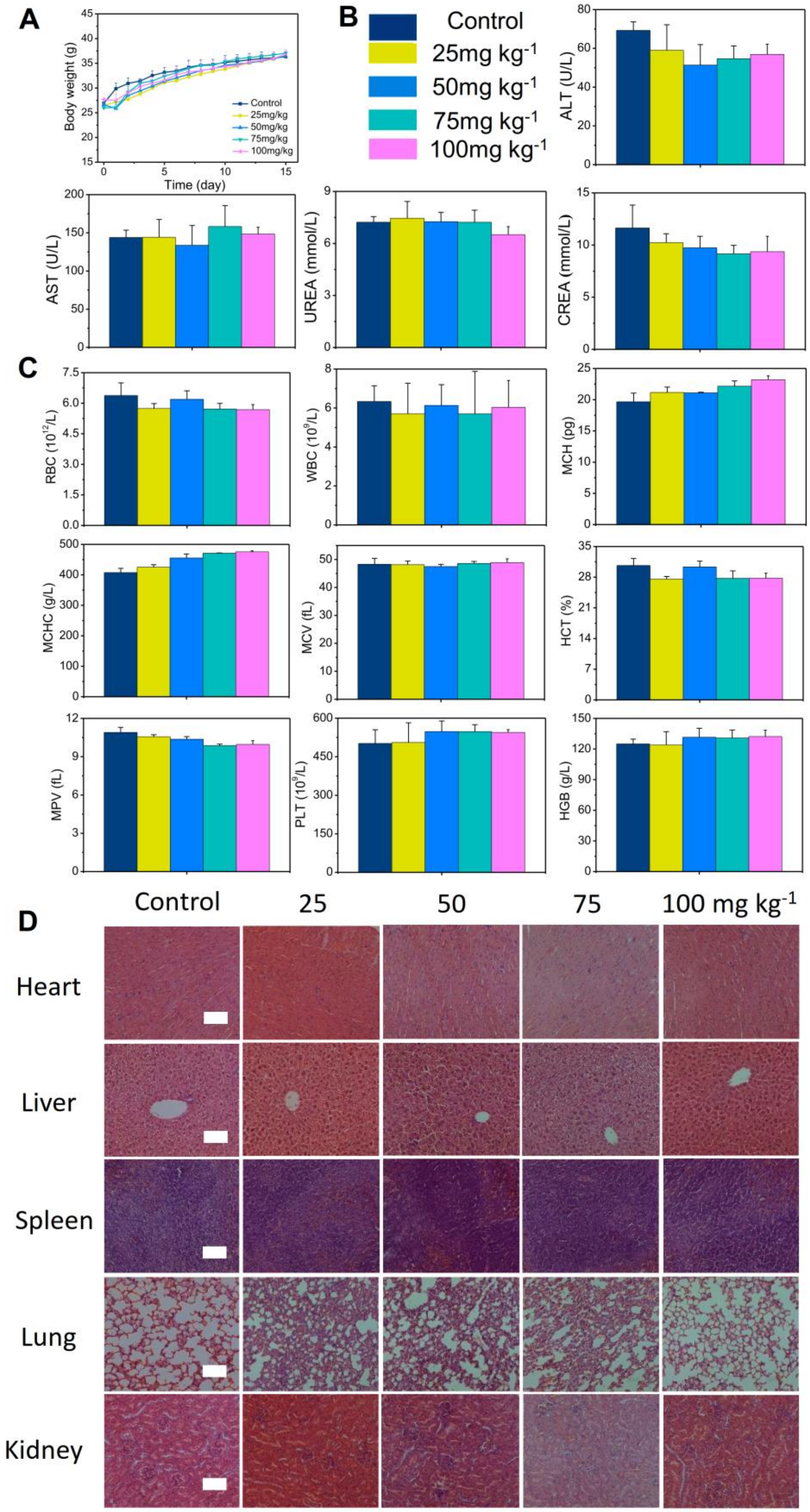

A
B
C
D
Control
25mg kg-1
50mg kg-1
75mg kg-1
100mg kg-1
Body weight (g)
Time (day)
25mg/kg
50mg/kg
75mg/kg
100mg/kg
ALT (U/L)
AST (U/L)
UREA (mmol/L)
CREA (mmol/L)
RBC (10^12/L)
WBC (10^9/L)
MCH (pg)
MCHC (g/L)
MCV (fL)
HCT (%)
MPV (fL)
PLT (10^9/L)
HGB (g/L)
Control
25
50
75
100 mg kg-1
Heart
Liver
Spleen
Lung
Kidney

Figure S3. Biocompatibility evaluation. (A) Changes in body weight in mice after intravenous administration of IQuCS@Zr-PEG NSPs at different concentrations. (B) Serum biochemical analysis in mice after intravenous administration of IQuCS@Zr-PEG NSPs at different concentrations results. (C) Routine blood examination results of mice injected with different concentrations of IQuCS@Zr-PEG NSPs intravenously. (D) Different concentrations of IQuCS@Zr-PEG NSPs after intravenous injection tissue H&E staining results. The scale bar is 50 μm.

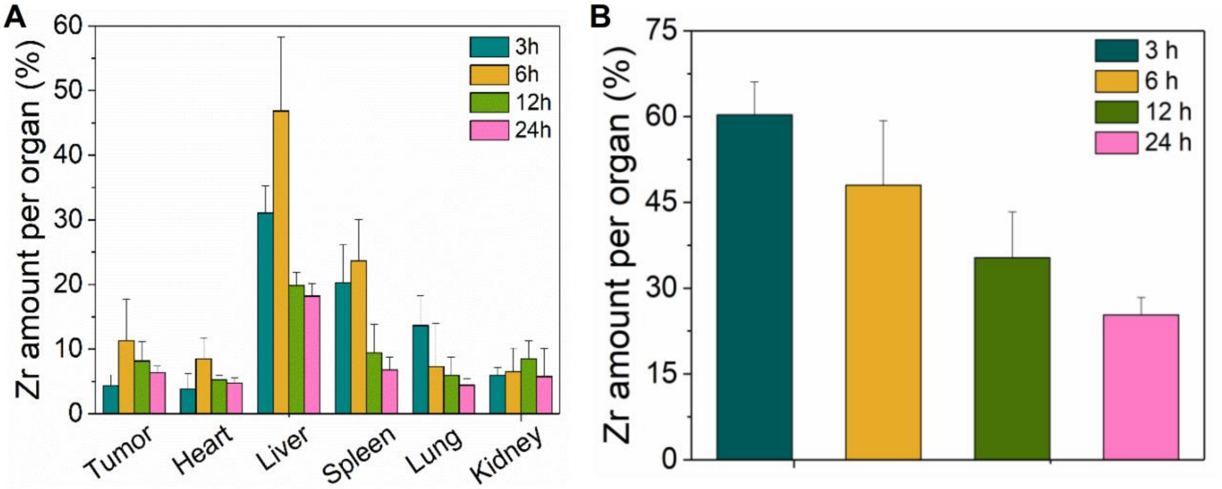

Figrue S4. Zr element distribution in vivo. (A) Distribution of Zr in tumors and major tissues from mice at 3 h, 6 h, 12 h and 24h after injection (the dose of IQuS@Zr-PEG NSPs was 50 mg/kg, n=3). (B) Distribution of Zr in blood from mice at 3 h, 6 h, 12 h and 24h after injection (the dose of IQuS@Zr-PEG NSPs was 50 mg/kg, n=3).

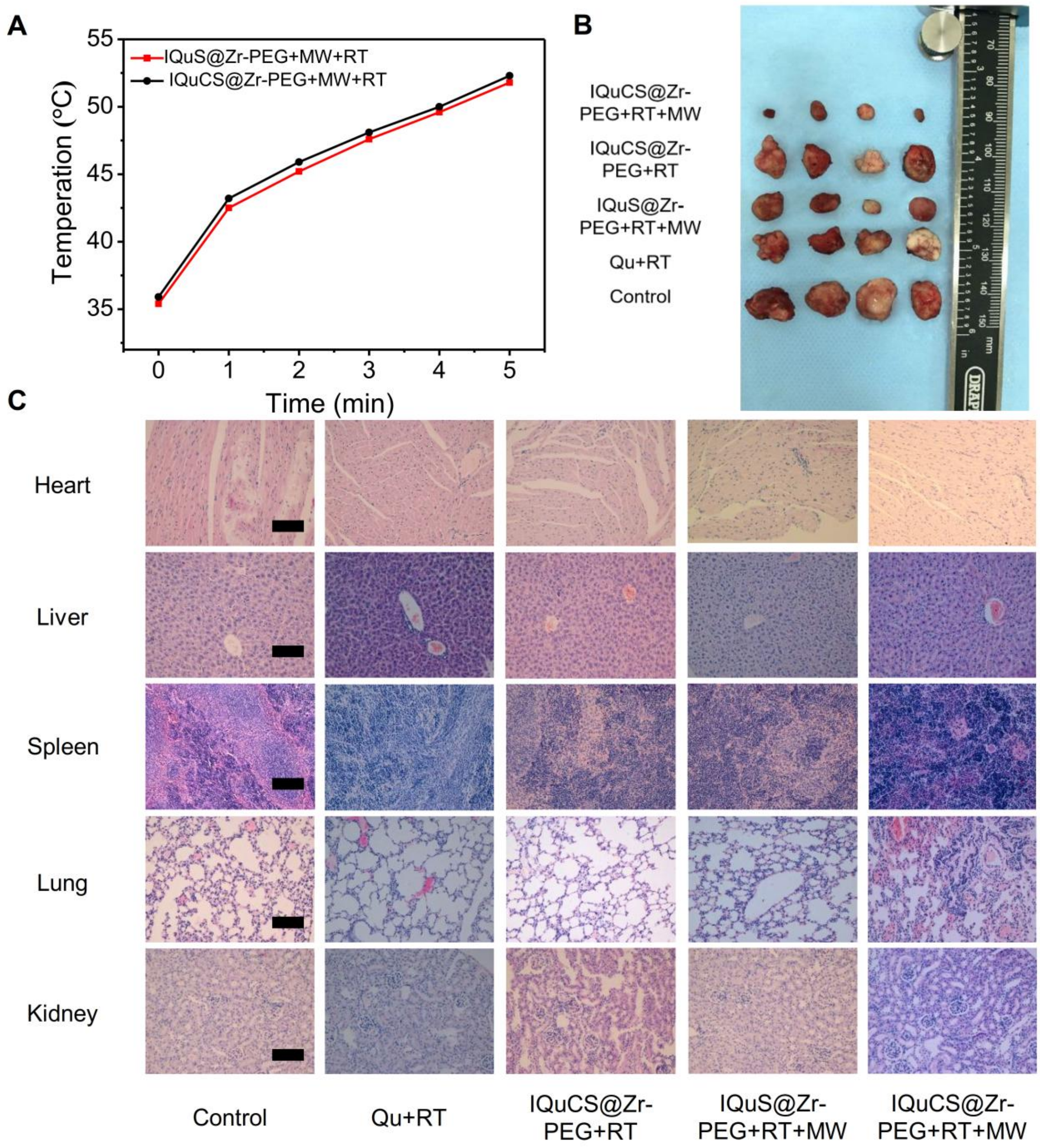

Figure S5. Evaluation of *in vivo* therapeutic experiments of IQuCS@Zr-PEG NSPs. (A) Temperature change curve per minute for treatment experiments. (B) Photographs of tumor tissues treated on the 14th day of each experimental mouse. (C) H&E staining sections of major organs after treatment for 14 days in each experimental mouse. The scale bar is 50 μm.

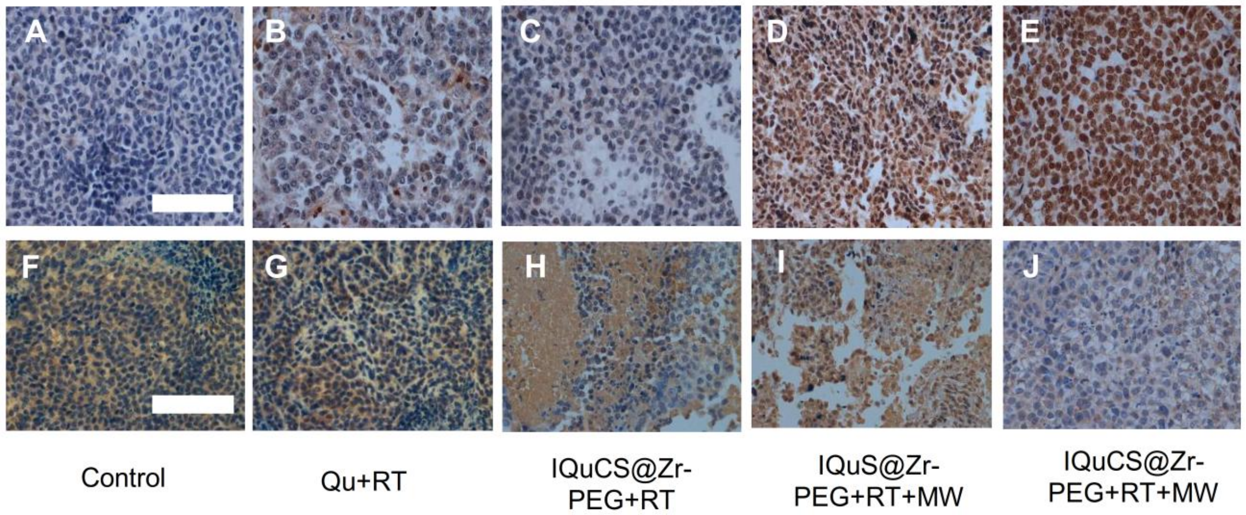

Figure S6. Experimental results of TUNEL and immunohistochemistry. (A-E) Figures A to E were the TUNEL results of the tumor tissues in the control group, Qu+RT group, IQuCS@Zr-PEG+RT group, IQuS@Zr-PEG+RT+MW group, and IQuCS@Zr-PEG+RT+MW group, respectively (magnification 400×). (F-J) Figures F to J were the immunohistochemistry results of the tumor tissues in the control group, Qu+RT group, IQuCS@Zr-PEG+RT group, IQuS@Zr-PEG+RT+MW group, and IQuCS@Zr-PEG+RT+MW group, respectively (magnification 400×). The scale bar is 50 μm.
